# Mechanistic Investigation into the Phase Separation Behavior of Soluplus in the Presence of Biorelevant Media

**DOI:** 10.1021/acs.molpharmaceut.4c01140

**Published:** 2025-03-11

**Authors:** Justus Johann Lange, Malte Bøgh Senniksen, Nicole Wyttenbach, Susanne Page, Lorraine M. Bateman, Patrick J. O’Dwyer, Wiebke Saal, Martin Kuentz, Brendan T. Griffin

**Affiliations:** † School of Pharmacy, University College Cork, College Road, Cork County, T12 R229 Cork , Ireland; ‡ Pharmaceutical R&D, F. Hoffmann-La Roche Ltd., Grenzacherstrasse 124, 4070 Basel, Switzerland; ¶ Fraunhofer Institute for Translational Medicine and Pharmacology, Theodor-Stern-Kai 7, 60596 Frankfurt am Main, Germany; § Roche Pharma Research and Early Development, Therapeutic Modalities, Roche Innovation Center Basel, F. Hoffmann-La Roche Ltd., Grenzacherstrasse 124, 4070 Basel, Switzerland; ∥ Analytical & Biological Research Facility, University College Cork, College Road, T12 YN60 Cork, Ireland; ⊥ Institute of Pharma Technology, University of Applied Sciences and Arts Northwestern Switzerland, Hofackerstrasse 30, CH-4132 Muttenz, Switzerland

**Keywords:** Soluplus, phase separation, amorphous solid dispersions, supersaturation, bioenabling formulations, solubility, liquid−liquid phase separation, formulation

## Abstract

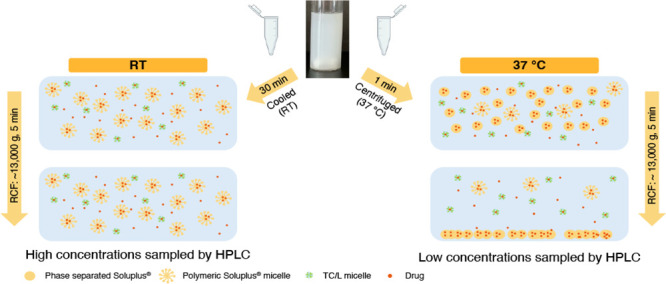

More than a decade since its introduction, the polymeric excipient Soluplus continues to receive considerable attention for its application in the development of amorphous solid dispersions (ASDs) and its utility as a solubilizer for drugs exhibiting solubility limited absorption. While it is well-recognized that Soluplus forms micelles, the impact of its lower critical solution temperature of approximately 40 °C remains an underexplored aspect. This study investigated the phase behavior of Soluplus in fasted-state simulated intestinal fluid (FaSSIF-V1). It was demonstrated that Soluplus forms a dispersed polymer-rich coacervate phase, which coexists with Soluplus micelles at 37 °C. This behavior was confirmed by cloud point measurements, visually discernible phases after centrifugation, as well as multi-angle dynamic light scattering (MADLS) measurements, and quantitative ^1^H-nuclear magnetic resonance (NMR) spectroscopy of Soluplus concentrations in the supernatant pre- and post-centrifugation. The practical relevance of these findings was contextualized by solvent shift experiments and dissolution testing of spray-dried ASD. The results demonstrated that the poorly water-soluble drug RO6897779 resided in a polymer-rich coacervate phase and was spun down during centrifugation, which resulted in an amorphous pellet exhibiting the characteristics of a viscous liquid. The entrapment of the drug within the polymer-rich phase was further analyzed by temperature- and time-dependent MADLS experiments. The findings of this study are of particular relevance for a mechanistic understanding, relevant to comprehending in vitro-in vivo relationships of Soluplus-based ASDs. Low sampled drug concentrations in FaSSIF-V1 at 37 °C may originate not only from limited drug release and precipitation but also from the formation of a drug-containing, polymer-rich Soluplus phase. Therefore, a liquid–liquid phase separation occurring from Soluplus-based formulations in a biorelevant medium can be excipient-driven, which is different from the common perception that phase separation in the solution state is triggered primarily by high drug concentrations exceeding their amorphous solubility.

## Introduction

Amorphous solid dispersions (ASDs) constitute one of the fundamental bioenabling formulation principles to address subtherapeutic concentrations of drugs demonstrating solubility limited absorption, which is clearly seen by a substantial increase in authorized ASD drug products since 2010.^1−5^ Aside from technical feasibility, the successful development of an ASD for drug candidates is contingent on physical and chemical formulation stability during shelf life, as well as sufficient dissolution and increased kinetic solubility enabled by supersaturation upon release.^6−8^ Such luminal supersaturation is accomplished by the amorphous characteristics of the drug, which eliminate the energy associated with crystal lattice disruption during the dissolution process.^9^ The amorphous form constitutes a thermodynamic high-free-energy state, which often requires suitable polymers to inhibit rapid nucleation and recrystallization of the drug. Another functionality of such polymers is to enhance supersaturation by improving dissolution rates and to maintain apparent supersaturation by acting as precipitation inhibitors through various mechanisms.^10,11^ Therefore, the selection of an appropriate polymeric carrier during formulation development is crucial to the successful development of an ASD.^11−13^

One of the more recently introduced polymers suited for ASD formulation development is Soluplus, a polyvinyl caprolactam-polyvinyl acetate-polyethylene glycol graft copolymer.^14,15^ Soluplus is increasingly utilized to address solubility limited absorption, as evidenced by numerous published research articles, by the clinical success in the form of approved generics in Europe, and by the application in investigational medicinal products.^15−19^ Among the many options of polymers currently used for the development of ASDs, Soluplus stands out due to its unique attributes, namely, its low glass transition temperature (*T*_g_) of approximately 70 °C, its amphiphilicity, which causes the formation of micelles, and its lower critical solution temperature (LCST) of about 40 °C in water.^20^

Above the LCST, a polymeric solution begins to separate into a two-phase system, composed of a polymer-rich coacervate phase and a polymer lean phase.^21,22^ This phase separation process is governed by the hydration of the polymer and may be influenced by ions and other additives that compete for water molecules during the hydration process.^23^ As a result of this, an unfavorable free energy of mixing may be induced, causing the system to phase-separate.^16^ This phenomenon becomes particularly complicated for copolymers composed of different monomers with varying solvation characteristics and those that show chemical heterogeneity, such as a broad polymer weight distribution.^22,24^

The LCST of a polymer is typically observed by tracing the cloud point (CP) of a polymer solution.^22,25^ This method involves starting with a homogeneous polymer–solvent solution and gradually increasing the temperature. At the LCST, the solution transitions from a single-phase to a two-phase system, where small droplets of the polymer-rich phase separate from the polymer-lean phase, causing the solution to become turbid. Inorganic salts are known to affect the clouding of polymers and proteins in solution due to alterations in their hydration.^26−30^ Hughey et al.^30^ conducted research on the CP of Soluplus solutions in the presence of inorganic salts and linked it to the dissolution behavior of corresponding ASDs. It was demonstrated that the variations in the polymer’s hydration, induced by the incorporated inorganic salt, influenced the tendency of carbamazepine ASDs to gel, erode, and ultimately release from the matrix. Similar trends were also observed for hydroxypropyl methylcellulose (HPMC)-based ASDs.^28,29^ The classical Hofmeister ion framework was applied to interpret hydration processes as promoted by chaotropic salts versus dehydration processes promoted by kosmotropic salts.^26^

Typically, biorelevant dissolution testing is conducted in simulated intestinal fluids to assess the formulation performance. These fluids include buffer salts, bile salts, and lecithin, and the tests are carried out at a temperature of 37 °C.^31^ A recent study by Niederquell et al.^32^ demonstrated a significant buffer effect during solvent shift experiments by studying the precipitation kinetics of four drugs in hydroxypropyl cellulose (HPC) solutions, in both bicarbonate and phosphate buffer. The study related buffer-dependent differences in precipitation kinetics to the salt-dependent hydration of HPC in solution and further substantiated hydration differences with full-atomistic molecular dynamics simulations. Similar experimental findings of supersaturation in alternative buffer types were also obtained by Jede et al.^33^ using a different polymer and set of drugs.

Pinto et al.^34^ investigated the influence of simulated intestinal media components, i.e., sodium taurocholate (Na-taurocholate) and lecithin on the precipitation kinetics of candesartan cilexetil in Soluplus solutions. Soluplus was shown to have a detrimental effect over neat fasted state simulated intestinal fluid (FaSSIF-V1) on precipitation, which is a critical finding considering Soluplus’ intended use as a precipitation inhibitor. These findings were related to interactions between lecithin and Soluplus, which caused an alteration in the microenvironmental polarity of micelles formed by the polymer.

Previous research efforts on the clouding behavior of Soluplus related their results to dissolution patterns of ASDs and described a medium or additive dependent relation between CP and the obtained results.^30,35^ However, the occurrence of phase separation once the CP or LCST is reached has not been investigated and is unique to Soluplus with its reported low LCST of about 40 °C that is close to body temperature.

The objective of this study was to explore the colloidal species formed by Soluplus under fasted state simulated intestinal conditions at 37 °C with an emphasis on Soluplus’ phase behavior. Furthermore, it was aimed to describe the implications this behavior may have for apparent supersaturation maintenance, as well as the analytical challenges related to sampling of such media.

## Materials and Methods

### Materials

RO6897779 was provided by Hoffmann-La Roche Ltd. (Basel, Switzerland). Soluplus (Lot: 96410247G0) was received from BASF (Ludwigshafen, Germany). NaH_2_PO_4_, NaCl, deionized water, acetonitrile, formic acid, and dimethyl sulfoxide (DMSO) were purchased from Sigma-Aldrich (Steinheim, Germany). NaOH was purchased from Merck KGaA (1N, Titripur, Darmstadt, Germany). 3F biorelevant media powder (Lot: BCCK8872) was procured from biorelevant.com LTD (London, United Kingdom). For quantitative ^1^H-Nuclear Magnetic Resonance spectroscopy (NMR) D_2_O (99.8 atom %D), (Thermo Scientific, Switzerland) and deuterated methanol (MeOD-*d*_4_) (99.8 atom %D) (SIGMA-Aldrich Co., MO, USA) were used. As ^1^H NMR standard 3-(Trimethylsilyl)propionic-2,2,3,3-d_4_ acid, sodium salt (TMSP-*d*_4_) (99.8 atom %D) (Aldrich Chemical Co., WI, USA) was employed. ^1^H NMR samples were submitted in Bruker SampleJet NMR tubes (*L* = 103.5 mm, O.D. 5.0 mm) with sealed caps. All solvents used for HPLC analysis were of an appropriate grade. *N*-Methyl-2-pyrrolidone (NMP) was purchased from VWR International (Rosny-sous-Bois Cedex, France), and N,N-dimethylacetamide was purchased from PanReac Applichem ITW reagents (Applichem GmbH, Darmstadt, Germany). Depending on the assay, either Eppendorf tubes (Eppendorf SE, Hamburg, Germany) or centrifugal filter units (Ultrafree - MC - HV) with a Durapore polyvinylidenefluoride (PVDF) (Merck Millipore Ltd., Tullagreen, Ireland) 0.45 μm porosity membrane were used for centrifugation. For multi-angle dynamic light scattering (MADLS), disposable cuvettes made of polystyrene (dimensions 10 × 4 × 45 mm) (Sarstedt AG & Co. KG, Nümbrecht, Germany) were employed.

### Properties of Model Drug RO6897779

**Figure 1:**
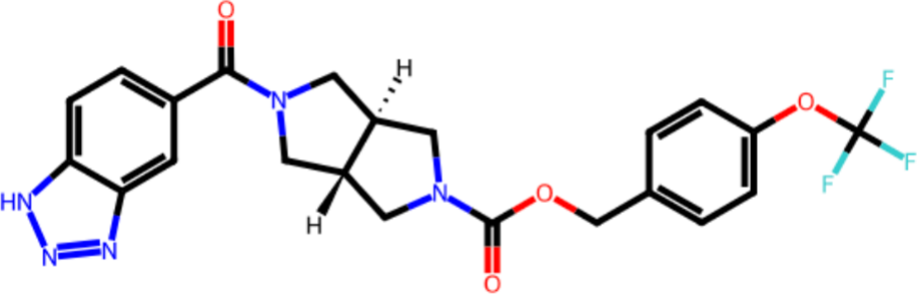
Molecular structure of RO6897779.

The physicochemical properties of the model compound RO6897779 are listed in Table 1, and the structural formula is depicted in Figure 1.

**Table 1:** Physicochemical Properties and Molecular Structure of RO6897779

property	value
molecular weight [g mol^–1^]	475.43
p*K*_a_ (acidic)^a^	8.25
melting point [°C]	183
glass transition temperature [°C]	91
*a*Log*P*^b^	4.15
crystallization tendency^c^	class III
solubility in FaSSIF-V1 [μg mL^–1^]^d^	22.90 (±1.11) (pH = 6.5 ± 0)

a Calculated p*K*_a_ by MoKa-software (Roche trained model, Molecular Discovery, Hertfordshire, UK).

b *a*Log*P* calculated by D360 Scientific Informatics Platform (Certara, Pennsylvania, US).

c Classification according to Baird et al.^36^

d RO6897779 Form I.

### Characterization of Different Soluplus Concentrations Dissolved in FaSSIF-V1 and Deionized Water

The Soluplus and RO6897779 concentrations used in the following sections are rationalized based on an anticipated dose of 250 mg and an ingested fluid volume of 250 mL. Based on that, drug-to-polymer ratios (D/P) of 10, 20, 30, and 40% corresponded to Soluplus concentrations of 9.00, 4.00, 2.33, and 1.50 mg mL^–1^ at a RO6897779 concentration of 1.00 mg mL^–1^ upon nominal release.

#### Cloud Point Determination

CP measurements were conducted to obtain an understanding of the LCST with increasing Soluplus concentrations when dissolved in FaSSIF-V1 and deionized water, respectively. A Chirascan Circular Dichroism Spectrometer was used (Applied Photophysics Limited, Surrey, UK) to measure the percentage of light transmission as a function of temperature. The wavelength of the instrument was set to 800 nm at a bandwidth of 1 nm. Light transmission was determined over a temperature range of 20 to 70 °C. The temperature was incrementally increased by 1.0 K min^–1^. Samples were loaded into a 10 mm quartz cuvette. An air background calibration with the same cuvette was carried out prior to analyzing each solution with three repeated measurements. The sample was equilibrated for 15 min at the starting temperature prior to measurement. The CP was determined at polymer concentrations of 9.00, 4.00, 2.33, and 1.50 mg mL^–1^, dissolved in FaSSIF-V1 and deionized water, respectively, to understand the influence of Soluplus concentration and biorelevant media components on the CP. The obtained sigmoidal transmission over temperature arrays were interpolated using SciPy^37^ to calculate the temperature at which 50% light transmission was observed, in line with the CP definition by Sarkar.^24^

#### Visual Assessment of Phase Separation

Centrifugation of Soluplus dissolved in FaSSIF-V1 at the concentrations outlined before was conducted to illustrate the extent to which Soluplus was phase-separated. Medium without any drug present was heated and stirred at 250 rpm in the microDISS Profiler (Pion Inc., Billerica, MA) using magnetic cross stirrer bars and equilibrated for 4 h at 37 °C. Subsequently, 10 mL of medium was centrifuged at a relative centrifugal force (RCF) of 3000 *g* at 37 °C for 5 min with an Eppendorf 5702R centrifuge (Hamburg, Germany) using Falcon Tubes (Sarstedt AG & Co. KG, Nümbrecht, Germany). The experiment was repeated at room temperature (RT) to verify the temperature influence on the extent of Soluplus phase separation. The experiments were repeated with another batch of Soluplus and biorelevant media to verify consistent behavior across batches.

#### ^1^H NMR Quantification of Soluplus

^1^H NMR spectroscopy analysis was conducted to quantitatively measure the amount of Soluplus phase separating. Thus, FaSSIF-V1 was prepared according to the protocol of biorelevant.com using D_2_O and adjusting pD to 6.91 as an appropriate correction factor for the isotope effect on pH/pD.^38,39^ Soluplus at the previously outlined concentrations was dissolved at RT and placed in an oven maintained at 37 °C (*n* = 3) (APT.lineTM BD (E2), Binder, GmbH, Tuttlingen, Germany) on a magnetic stirring unit (Mixdrive 15, 2MAG, München, Germany) at 300 rpm. The temperature was externally verified to be at 37 °C. For sample preparation, 500 μL aliquots were withdrawn and centrifuged through Ultrafree centrifugal units at 11,500 *g* for 5 min at 37 °C (Mikro 200 R, Hettich Zentrifugen GmbH, Tuttlingen, Germany). Subsequently, 300 μL was withdrawn and diluted 1:1 [v/v] with a stock solution of MeOD-*d*_4_ containing a known amount of TMSP-*d*_4_ as an internal reference standard. The same procedure was repeated with uncentrifuged media.

To establish a calibration curve for quantitative ^1^H NMR, the same MeOD-*d*_4_ solution containing TMSP-*d*_4_ was used. Soluplus solutions in deuterated FaSSIF-V1 were prepared and covered concentrations in the range of ≈0.05 to 5 mg mL^–1^. The integral ratio of the PEG6000 signal to the internal standard was used to establish a linear calibration curve for sample quantification. The calibration curve and experiments were conducted with the same batch of methanol and FaSSIF-V1 and were prepared with the same solvent mixture (1:1) [v/v].

^1^H NMR acquisitions were conducted using a Bruker Avance III 600 MHz Ultrashield NMR Spectrometer (Coventry, UK) fitted with a triple channel inverse (TCI) 5 mm cryoprobe. The zg pulse program was selected, and scans were acquired with a relaxation delay of 30 s. 131,072 data points were selected to ensure high spectral resolution. The samples were maintained at 298 K. Spectral acquisition, processing, and analysis were conducted through the software TopSpin V4.3.0.

### Solubility Measurements of Crystalline RO6897779

The solubility of RO6897779 was determined in FaSSIF-V1 and in the presence of previously outlined Soluplus concentrations dissolved in FaSSIF-V1 to determine the apparent degree of supersaturation induced during solvent shift experiments. Samples were prepared by adding an excess amount of RO6897779 (≈5 mg) to 3 mL of the excipient solutions, or neat FaSSIF-V1 medium, and were stirred at 250 rpm at 37 °C (Mixdrive 15, 2MAG, München, Germany). Samples were equilibrated for 48 h. Solid–liquid separation was conducted by centrifugation at 11,500 *g* for 5 min using Ultrafree centrifugal units. The supernatant was immediately diluted with a 1:1 (v/v) mixture of acetonitrile and deionized water to fall into the linear region of the calibration curve during high-performance liquid chromatography (HPLC), as described under HPLC drug quantification below.

### Supersaturation Kinetics of RO6897779 in the Presence of Soluplus

Solvent shift experiments were conducted in the Pion microDISS Profiler to assess the supersaturation kinetics of RO6897779 in the presence of Soluplus dissolved in FaSSIF-V1. The clouding behavior of Soluplus at 37 °C did not permit usage of in-line UV analysis; hence, offline HPLC analysis was employed for drug quantification. Supersaturation kinetics were assessed at D/P ratios of 10, 20, 30, and 40% corresponding to the polymer concentrations outlined previously. The polymer was dissolved in FaSSIF-V1 at RT and subsequently heated to 37 °C. A concentrated DMSO stock solution was prepared at a concentration of 50 mg mL^–1^ RO6897779. From this stock solution, an aliquot of 400 μL was spiked into 19.6 mL of preheated solvent shift medium, so that a nominal concentration of 1 mg mL^–1^ RO6897779 was obtained without exceeding DMSO concentrations of 2% [v/v], while assuming a negligible residual solvent influence on solubility.^40^ The solutions were stirred at 250 rpm and the temperature was maintained at 37 °C and continuously monitored. At time points of 5, 15, 30, 60, 120, 180, and 240 min, 2 × 200 μL aliquots were withdrawn. Preliminary studies indicated that Soluplus phase-separates temperature-reversibly. Hence, to investigate drug incorporation in such phase, samples were either immediately centrifuged or cooled to RT within 30 min before centrifugation at 13,684 *g* (Mikro 120, Andreas Hettich GmbH & Co. KG, Germany) in Eppendorf tubes (Eppendorf, Germany) for 5 min. Subsequently, the supernatant of the media was diluted appropriately with an acetonitrile–water mixture. An intermission of 30 min was introduced as a change in Soluplus phase behavior was expected once the sample reached RT. It was assumed that this intermission would lead to a difference in the sampled concentration of RO6897779.

### Preparation of Amorphous Solid Dispersion Material by Spray Drying

A binary amorphous solid dispersion of RO6897779 with the polymeric carrier Soluplus was prepared by spray drying using a Büchi Mini Spray Dryer B-290 coupled with an Inert Loop B-295 and Dehumidifier B-296 (Büchi, Flawil, Switzerland). The spray dryer was equipped with a 1.5 mm 2-fluid nozzle utilizing a 414 L h^–1^ atomizing nitrogen flow rate, a high-efficiency cyclone, an aspirator set to maximal performance 35 m^3^ h^–1^ and a condenser set to −20 °C. Spray drying was carried out using a solution of RO6897779 and Soluplus dissolved in methanol, with a solid content of 5% [w/v], using a peristaltic pump set to pump at a rate of 6 g min^–1^. The solid load was not increased due to the limited solubility of RO6897779 in organic solvents. A drug load of 10% [w/w] was selected for spray drying based on results from preliminary investigations. An inlet temperature of 75 °C for the nitrogen drying gas was used to ensure immediate evaporation of methanol without exceeding the glass transition temperature of the solids to mitigate material sticking to the walls of the heating chamber. This inlet temperature resulted in an outlet temperature of 34 °C. After spray drying, the prepared material was immediately transferred to a vacuum oven set to 40 °C under a 20 mbar vacuum and dried overnight to remove residual solvent. The material was stored at 5 °C over a desiccant until further use.

The drug content of the ASD material was determined by dissolving 5 mg of the spray-dried powder in 10 mL of NMP and subsequent UPLC quantification. The loading efficiency (LE) was calculated as outlined in eq 1
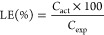
1where *C*_act_ is the measured concentration in the dissolved powder sample and *C*_exp_ denotes the theoretically expected drug concentration of the sample.

### Preparation of the Physical Mixture Material

A physical mixture of Soluplus and RO6897779 at a drug load of 10% [w/w] was prepared. Due to particle size differences between the drug and the excipient, Soluplus was milled and subsequently combined and mixed with RO6897779. A Retsch MM 200 ball mill (Retsch GmbH, Haan, Germany) was used and fitted with a 20 mL stainless steel custom-made milling chamber. Approximately 10 g of Soluplus was dispensed into the milling chamber, and three stainless steel beads (*D* = 10 mm) were added. The material was milled within three iterations at 25 Hz. A two-minute intermission was introduced between the iterations to avoid overheating of the material. Subsequently, the material was sieved through a 180 μm sieve to obtain a homogeneous particle size distribution. The material was combined with micronized RO6897779 at a drug load of 10% (w/w) and dispensed into a glass vial. The same vial was mounted onto the Retsch mill excluding the stainless steel beads, and a mixing step of 5 min at 10 Hz was applied. The drug content of the physical mixture was determined in triplicate by sampling 10 mg, each after mildly agitating the vial and dissolving the material in 1 mL of methanol. The samples were diluted 20-fold and RO6897779 was quantified via HPLC analysis.

### Powder Dissolution of Binary Amorphous Solid Dispersion and Physical Mixture

Non-sink dissolution experiments were conducted using the microDISS Profiler in FaSSIF-V1 at 37 °C for 10% DL [w/w] Soluplus-based spray-dried dispersion (SDD) material of RO6897779. A nominal concentration of 1 mg mL^–1^ of RO6897779 was targeted by dispensing 200 mg of the 10% [w/w] drug load SDD into 20 mL of preheated FaSSIF-V1 in triplicate. A wetting step was introduced by stirring at 600 rpm for 1 min at the beginning of the dissolution experiment, to agitate the powder bed and promote dispersion. The experiment was conducted over a period of 20 h to achieve complete dissolution of the spray-dried dispersion, with the aim to fully observe the hypothesized phase separation behavior. The same dissolution protocol was applied to the physical mixture.

### HPLC Drug Quantification

RO6897779 was quantified using reversed-phase HPLC. An Agilent 1200 series HPLC system (Agilent Technologies Inc., US) equipped with an Agilent Eclipse Plus C18 column (5 μm, 4.6 mm × 150 mm) was used for sample separation. Detection and quantification of RO6897779 were performed by using a variable wavelength detector set to 214 nm. The mobile phase consisted of (A) HPLC-grade deionized water and (B) and acetonitrile both containing 0.1% [v/v] formic acid. Each injection had a runtime of 19 min. Solvent B was linearly increased from 10 to 90% over 15 min, followed by a return to 10% within 3 min, maintaining an isocratic flow for one minute before the subsequent injection. The retention time of RO6897779 was observed at 11.44 min, with an injection volume of 10 μL, using a flow rate of 1 mL min^–1^. Data analysis was conducted using Agilent’s OpenLab CDS software Version 2.6.

### Solid State Analysis via X-ray Powder Diffraction

X-ray powder diffraction (XRPD) was conducted under ambient temperature, employing an STOE STADI-MP diffractometer (STOE and Cie GmbH, Darmstadt, Germany). After the 4 h solvent shift experiment, the media was centrifuged at 13,684 *g*. Spun-down material was isolated by decanting the supernatant, then removed with a spatula and distributed on cellulose acetate transmission foils. The diffractometer was set to transmission mode with a linear PSD detector. The anode current was set to 40 mA and the anode voltage to 40 kV. X-radiation was induced with a Cu *K*α1 (λ = 1.5046 Å) source and a Germanium monochromator. The 2θ range was 3.5 to 36° in scanning steps of 1° per 90 s. The samples were prepared and analyzed immediately after the solvent shift experiment. A diffractogram of the crystalline reference material and prepared ASDs was obtained with the same instrument settings.

### Differential Scanning Calorimetry

The manufactured spray-dried dispersion as well as the neat crystalline drug was investigated in triplicate using DSC to detect the presence of crystalline material and to gain information about the glass transition temperature (*T*_g_) of the materials. For this purpose, a DSC 1 Star system instrument from Mettler-Toledo AG (Greifensee, Switzerland) was used. The instrument was calibrated for temperature and heat flow using indium, and the analysis was performed with an air flow rate of 150 mL min^–1^. Samples were weighed into 40 μL aluminum pans (Mettler-Toledo AG, Greifensee, CH), sealed with pierced aluminum lids, and placed into the DSC. The analysis of the spray-dried dispersion was carried out as a heating cycle which heated the sample from RT to 190 °C at 10 K min^–1^, followed by cooling down to −50 °C at −20 K min^–1^ and finally reheating to 190 °C at 10 K min^–1^. The analysis of the neat crystalline drug was carried out according to Baird et al.,^36^ to evaluate the glass forming ability of the drug. The second heating ramp was used to measure the *T*_g_ without the influence of evaporating moisture.

### Headspace Gas Chromatography

Residual solvent in the spray-dried dispersion was determined using an Agilent 7890B Gas Chromatograph (Agilent Technologies, Palo Alto, CA, USA) with an Agilent 7697A Headspace Autosampler (Agilent Technologies, Palo Alto, CA, USA) fitted with a flame ionization detector (FID). Weighed samples of approximately 20 mg each were dissolved in 1 mL of N,N-dimethylacetamide and incubated at 85 °C for 10 min before injection. An Agilent DB-624 (60 m, 0.25 mm, 1.4 μm) column was used for chromatographic separation. The FID temperature was set at 250 °C and the total run time was 40 min. The LOQ reporting limit is 0.1% (1000 ppm).

### Scanning Electron Microscopy

The morphology of the spray-dried particles was investigated by using a TM3000 tabletop scanning electron microscope (SEM) (Hitachi High-Technologies Corporation, Krefeld, Germany). Samples were prepared by gentle deposition of ASD material (≤1 mg) onto the surface of a specimen stub covered with double-sided conductive tape. Any excess particles that were not entirely adhered to the conductive tape were gently shaken off prior to imaging. The samples were evaluated at magnifications of 500–2000 fold under high vacuum, with the accelerating voltage of the electron beam set to 15 kV.

### Multi Angle Dynamic Light Scattering Analysis of Colloidal Species

Multi-angle dynamic light scattering (MADLS), conducted through the ZetaSizer Ultra instrument (Malvern Panalytical, Malvern, United Kingdom) was employed to analyze particle size distributions. Size analysis was conducted after the solvent shift experiments at different D/P ratios and temperatures, as well as pre- and post-centrifugation. MADLS was chosen for its capability to discern individual particle size distributions of multicomponent systems to provide more reliability in the particle size measured. Disposable cuvettes were loaded with 500 μL of solvent shift media. Samples were either centrifuged for 5 min at 13,684 *g* under ambient conditions or directly analyzed after sampling from the solvent shift media. The data are reported as particle size by intensity. Samples were equilibrated for 10 min at the targeted temperature before analyzing the sample in triplicate. The dispersant was set to water with a refractive index of 1.33 and viscosity of 0.8872 mPa s.

## Results

### Characterization of Increasing Soluplus Concentrations in Solution State

#### Cloud Point Determination

**Figure 2:**
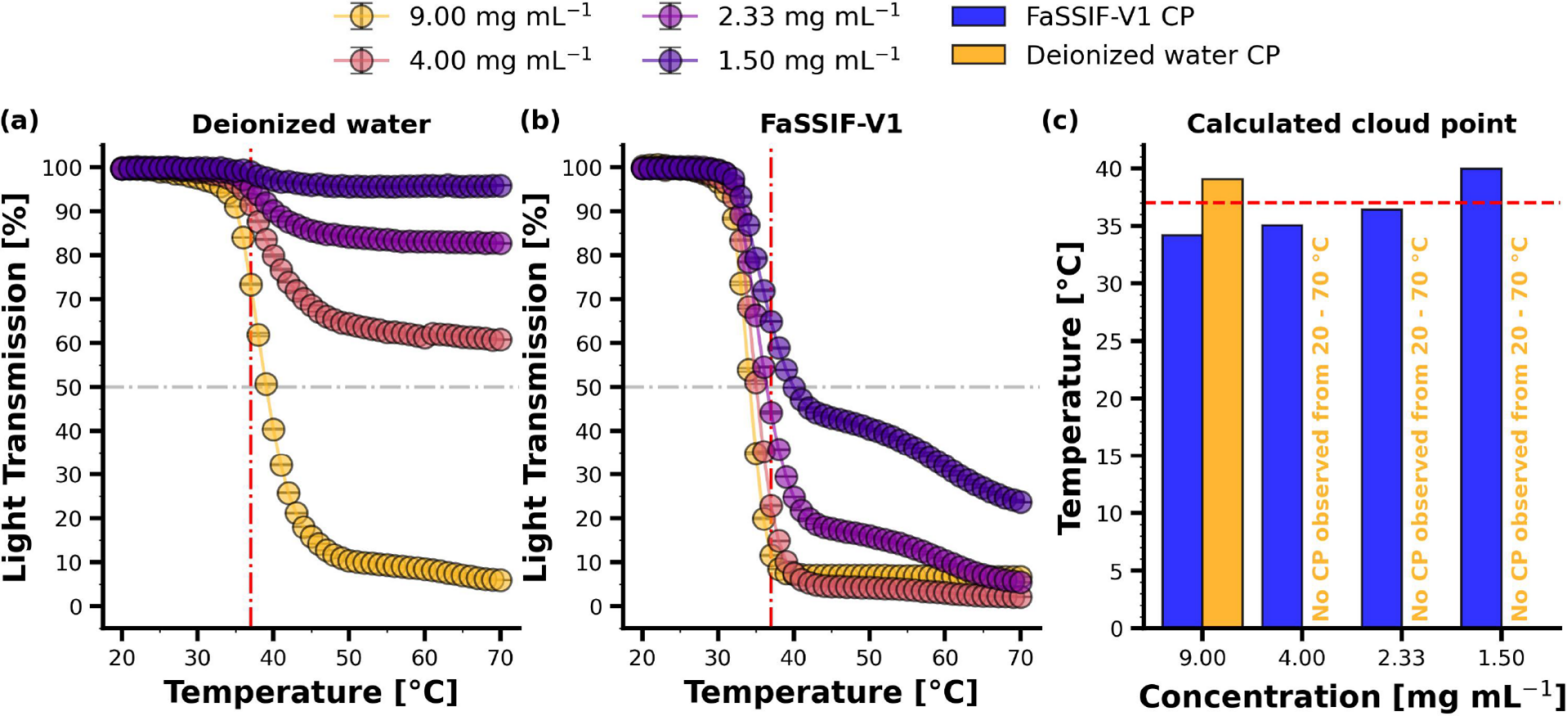
Sigmoidal light transmission curve as a function of temperature for different Soluplus concentrations dissolved in (a) deionized water and (b) FaSSIF-V1. The red dash dotted line denotes 37 °C and the gray dash dotted line denotes the cloud point (CP) (50% light transmission). Figure (c) summarizes the calculated CPs in either FaSSIF-V1 or deionized water. A numerical summary of the calculated cloud points can be inferred from Table S1 in the SI.

The clouding behavior of Soluplus was investigated at different polymer concentrations in deionized water and FaSSIF-V1. The light transmission over temperature profiles is depicted in Figure 2.

Figure 2a presents the sigmoidal light transmission profiles for the different Soluplus concentrations dissolved in deionized water. The magnitude of the decrease in light transmission was highly dependent on polymer concentration. For the highest Soluplus concentration studied, i.e., 9.00 mg mL^–1^, the cloud point was determined as 39.07 °C, whereas for more diluted polymer solutions, i.e., ≤4.00 mg mL^–1^, a cloud point was not detected, which is reflected by a less pronounced decrease in light transmission.

The light transmission over temperature profiles for Soluplus dissolved in FaSSIF-V1 are depicted in Figure 2b. For polymer concentrations of 9.00 and 4.00 mg mL^–1^ sigmoidally shaped curves were obtained, which approached 0% light transmission by the end of the temperature ramp. For concentrations of 2.33 and 1.50 mg mL^–1^, the decrease in light transmission was less steep and did not reach similarly low values compared to the higher polymer concentrations. Although the CP was not determined to be below 37 °C for solutions containing 1.50 mg mL^–1^ Soluplus, a substantial decrease in light transmission was observed at this temperature. Overall, a considerable decrease in light transmission was noted in FaSSIF-V1, with cloud points determined in proximity to body temperature. The results demonstrate a non-linear dependency between the decrease in light transmission and polymer concentrations, as illustrated in Figure S1 in the Supporting Information (SI). Biorelevant media components influenced the CP and decreased it to lower values for all polymer concentrations investigated. At polymer concentrations of ≥2.33 mg mL^–1^, the CP was determined to be below 37 °C. A numerical summary of the cloud points is presented in Table S1 (SI).

#### Visual Assessment of Phase Separation

**Figure 3:**
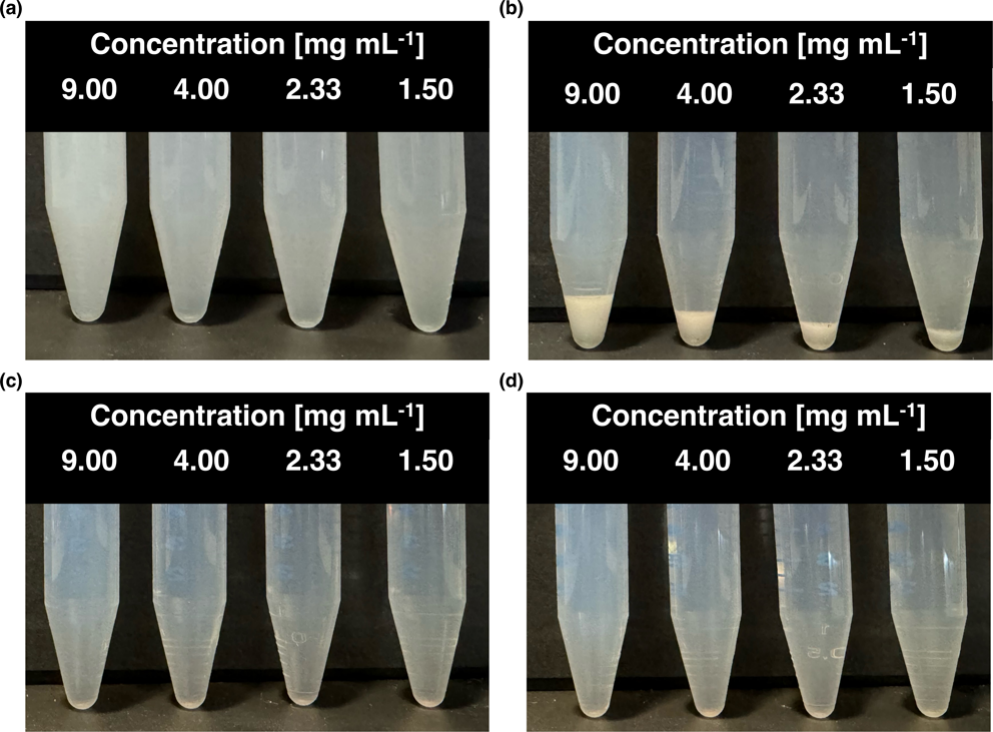
Polymer solutions at 37 °C and RT pre- and post-centrifugation. (a) Pre-centrifugation at 37 °C, (b) post-centrifugation at 37 °C, (c) pre-centrifugation at RT, (d) post-centrifugation at RT. Samples were centrifuged at 3000 *g* for 5 min.

The visual characteristics of Soluplus at different polymer concentrations dissolved in FaSSIF-V1 are illustrated in Figure 3 before and after sample centrifugation at 37 °C for 5 min at 3000 *g*.

The samples’ appearances before centrifugation substantiate the previously characterized clouding behavior of Soluplus in FaSSIF-V1 as can be observed when comparing the solutions at 37 °C against the same samples at RT (Figure 3a vs c).

Clouding behavior was observed across all polymer concentrations at 37 °C. Notably, even at a concentration of 1.50 mg mL^–1^, where the CP was calculated to be higher than 37 °C (Figure 2), the sample demonstrated signs of increased turbidity (Figure 3a). While according to Sarkar^24^ the CP was technically not reached, the visual characteristics indicate a considerably reduced light transmission which was reflected in the light transmission measurements. Visually, the different samples displayed only minor differences in turbidity, with only the solutions containing 9.00 and 4.00 mg mL^–1^ demonstrating a marginally higher degree of turbidity, confirming a concentration dependence between CP and polymer concentration.

After centrifugation of the samples at 37 °C, a visually evident pellet was obtained in all cases, as illustrated in Figure 3b. Additionally, the supernatant of each sample appeared clearer compared to the uncentrifuged samples, indicating that the fraction of the polymer responsible for the clouding behavior was spun down by centrifugation. After cooling to RT, the pellet changed its appearance to a translucent state (Figure S2 (SI)). The spun-down phase exhibited liquid characteristics and readily went into solution again by agitation after cooling it down to RT. The samples maintained at RT demonstrated the characteristic opalescence for Soluplus. There were no macroscopic signs of spun-down material observable.

#### Multi-Angle Dynamic Light Scattering of Soluplus Pre- and Post-centrifugation

**Figure 4:**
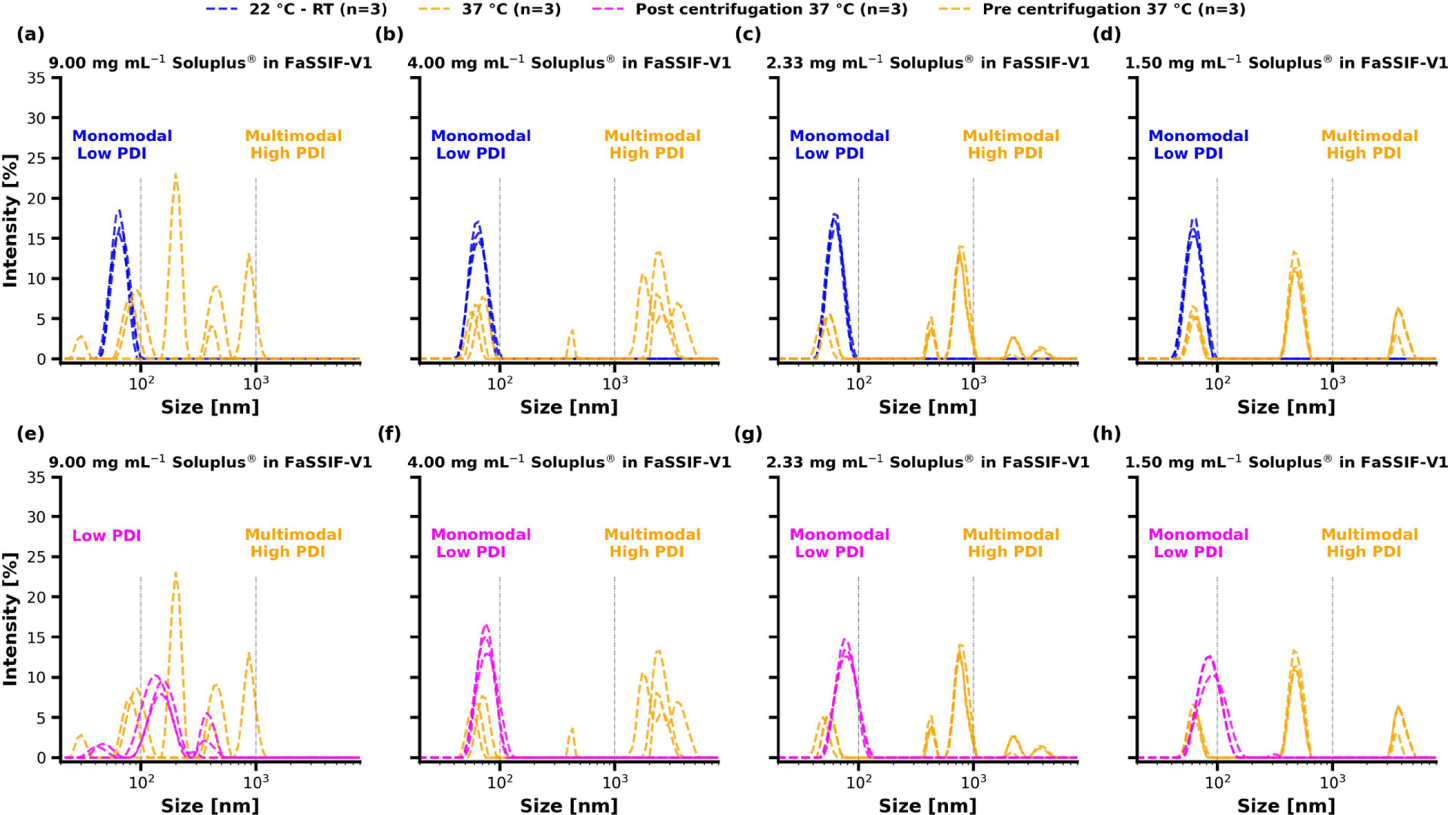
Size by intensity distributions of Soluplus determined by multi-angle dynamic light scattering (MADLS) at (a–d) room temperature (RT) and 37 °C, and (e–h) at 37 °C pre- and post-centrifugation. The samples subjected to centrifugation were centrifuged through a 0.45 μm filter membrane at 37 °C for 5 min at 11,500 *g*.

Soluplus was further assessed by MADLS at RT and 37 °C pre- and post-centrifugation to investigate the colloidal species present in the polymer solution at different conditions. As illustrated in Figure 4a–d, temperature had a pronounced influence on the colloidal size distributions of Soluplus.

The polymer’s size distribution at RT was monomodal at a size of approximately 60–70 nm across all polymer concentrations investigated. Soluplus micelles and those formed by Na-taurocholate and lecithin are known to overlap as reported by Schlauersbach et al.^39^ Increasing the temperature to 37 °C resulted in a high polydispersity, reflecting multimodal size distributions. Due to the high polydispersity, the presented data can only be assessed qualitatively. However, the data demonstrated that highly polydisperse colloidal distributions were detected. Mixed micelles of sodium taurocholate and lecithin did not substantially increase in size upon a temperature increase (data not shown).

Figure 4e–h illustrates the samples before and after centrifugation for 5 min at 11,500 *g* through a filter membrane of 0.45 μm porosity. After centrifugation, the size distributions demonstrated a monomodal size distribution with the exception of polymer concentrations of 9.00 mg mL^–1^, where only a lower degree of multimodality was noted compared with the same sample before centrifugation. Centrifugation influenced the quantified particle size populations substantially. Due to the visual characteristics reported in Figure 3, the change in size distribution is likely to be caused by a separation of phase-separated Soluplus by centrifugation.

#### ^1^H NMR Quantification of Soluplus Pre- and Post-centrifugation

**Figure 5:**
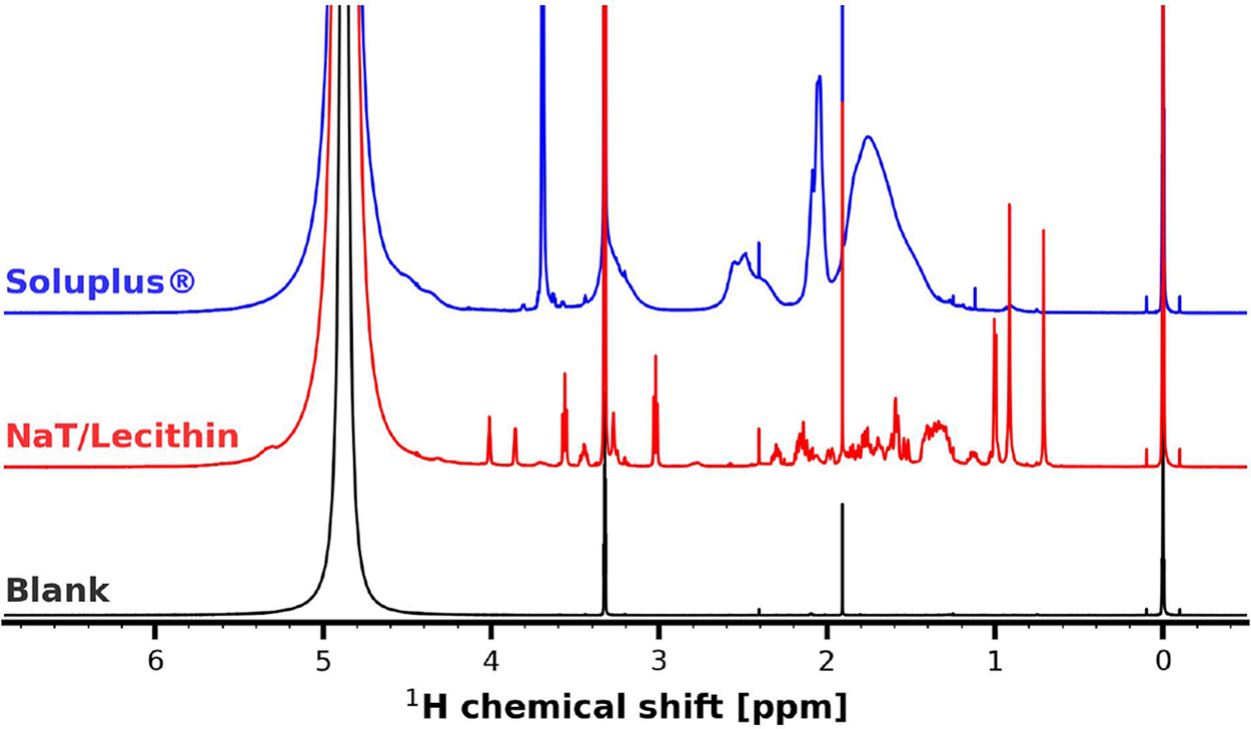
Solution ^1^H NMR spectra of Soluplus, sodium taurocholate (NaT)/lecithin and the blank solvent mixture used for analysis. Protons at 3.69 ppm, attributable to the PEG6000 moiety of Soluplus were used for quantification. The solvent mixture used for analysis was comprised of D_2_O and MeOD-*d*_4_ 1:1 [v/v], as well as TMSP-*d*_4_ (0 ppm) as the internal reference standard.

To quantitatively assess the extent to which Soluplus is spun down during centrifugation, quantitative ^1^H NMR was employed. Signals were assigned according to the studies of Sofroniou et al.^41^ The relative integration of PEG6000 at 3.69 ppm to that of the internal standard TMSP-*d*_4_ (0 ppm) was correlated against polymer concentration. Spectral information can be obtained from Figure 5.

**Figure 6:**
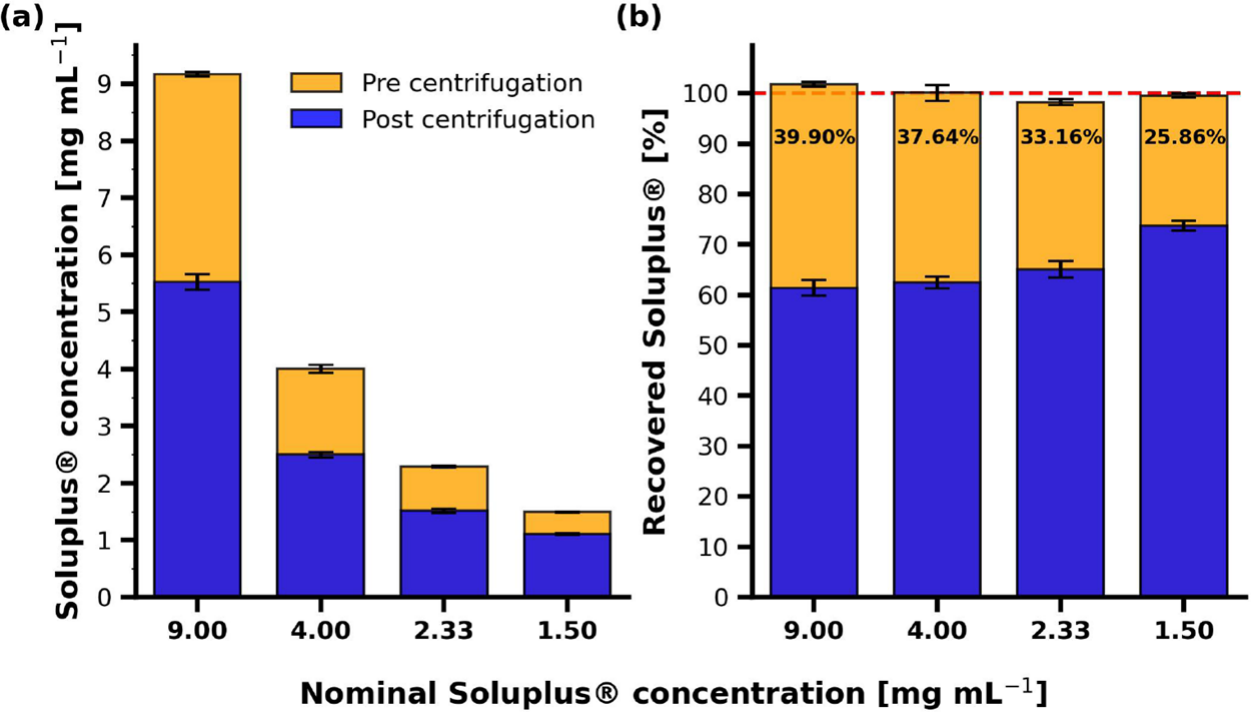
Quantification of Soluplus before and after centrifugation via ^1^H NMR. (a) Results presented as total amount of Soluplus before and after centrifugation. (b) Results illustrated as percentage Soluplus before and after centrifugation. The latter results are normalized by their nominal concentration. The samples were centrifuged at 37 °C for 5 min at 11,500 *g* through a filter membrane of 0.45 μm porosity (PVDF).

A linear correlation was established, and the calibration curve is displayed in Figure S4 (SI). Figure 6 presents the quantitative NMR results obtained for Soluplus dissolved in FaSSIF-V1 before and after centrifugation. ^1^H NMR successfully quantified Soluplus as evidenced by the accurate recovery of Soluplus before centrifugation (Figure 6). The concentration of Soluplus remaining in the supernatant after centrifugation differed depending on the polymer concentration employed, and no concentration-independent equilibrium value was observed.

To assess the fraction of Soluplus spun down by centrifugation, the nominal concentration of Soluplus was set to 100% and the spun-down amount was calculated accordingly. Figure 6b presents these relative ratios. The highest relative amount of Soluplus was spun down for the sample containing 9.00 mg mL^–1^ Soluplus and decreased with decreasing polymer concentration.

During centrifugation for the ^1^H NMR sample preparation, it was noted that Soluplus escaped the filter of the centrifugal filter unit as a white, viscous pellet was forming at the bottom of the tube, which indicates liquid-like characteristics of the polymer and/or particle sizes below 450 nm.

### Drug Incorporation in Phase-Separated Soluplus at 37 °C in FaSSIF-V1

#### Solubility Determination of Crystalline RO6897779

The solubility of RO6897779 was determined after 48 h of equilibration at 37 °C. The results are depicted in Table 2. XRPD analysis of the residual solids after equilibration did not demonstrate any polymorphic form changes (Figure S6).

**Table 2:** Solubility of RO6897779 Form I at Different Polymer Concentrations after 48 of Equilibration at 37 °C

no.	D/P [%]	soluplus [mg mL^–1^]	solubility [μg mL^–1^]
1	10	9.00	215.04 (±23.79)
2	20	4.00	130.11 (±13.83)
3	30	2.33	96.17 (±1.84)
4	40	1.50	68.97 (±2.74)
5	100	0.00	22.90 (±1.11)

#### Supersaturation Kinetics of RO6897779 in the Presence of Soluplus

**Figure 7:**
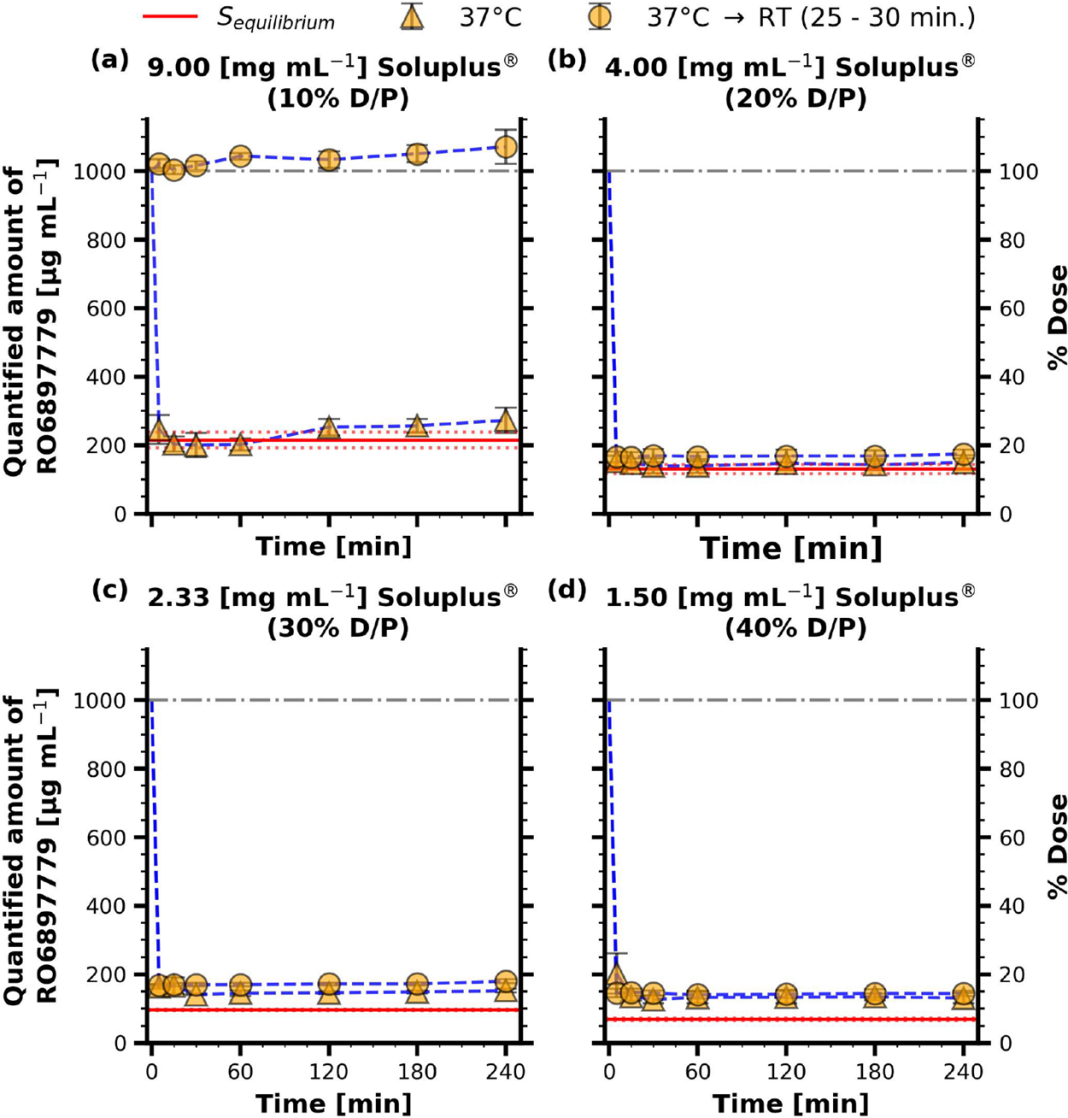
Supersaturation kinetics of RO6897779 in the presence of different Soluplus concentrations dissolved in FaSSIF-V1. The samples denoted as 37 °C → RT were cooled to room temperature (RT) within 30 min before centrifugation. Samples denoted as 37 °C were centrifuged immediately. The red lines correspond to the equilibrium solubility of Form I of RO6897779 in each system.

The supersaturation kinetics of RO6897779 in the presence of different Soluplus concentrations dissolved in FaSSIF-V1 were investigated by means of solvent shift experiments. Samples were either centrifuged immediately after withdrawing aliquots from the solvent shift media or cooled to RT within 30 min prior to centrifugation. The results are depicted in Figure 7.

**Figure 8:**
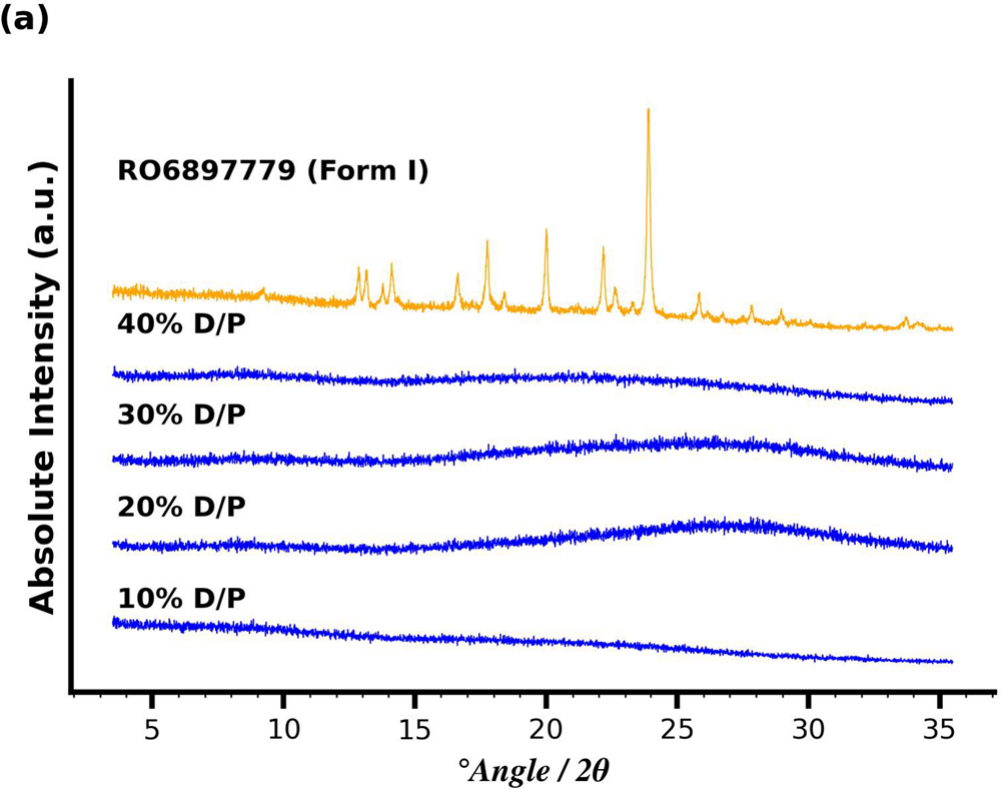
Solid state characteristics of spun down material via centrifugation after the four hour solvent shift experiment. RO6897779 Form I is included as the reference spectrum.

At a D/P ratio of 10% (Figure 7a), an apparent degree of supersaturation (aDS) of 4.65 was induced. Immediate sample centrifugation at 37 °C resulted in a substantial reduction in kinetic solubility values, suggesting a rapid onset of precipitation. Throughout the triplicate measurement, it was not possible to detect any birefringent material by polarized light microscopy (PLM). However, a pellet was recovered after centrifugation, which was analyzed by XRPD. During sample preparation, it was evident that the pellet was a highly viscous phase, which resembled hydrated Soluplus and did not consist of separate particles. This viscous phase was spread on cellulose acetate foils and submitted to XRPD. The pellet was XRPD amorphous, as depicted in Figure 8. A pattern of the crystalline material (RO6897779 Form I) is provided for reference.

For the second batch of samples taken from the solvent shift media at the same time points, there was an intermission of 25 to 30 min to allow the samples to cool down to RT. Due to the temperature reversibility of the clouding behavior, the solutions appeared clear and opalescent, rather than cloudy. Subsequent dilution and quantification resulted in sampling of nominal drug concentrations, and it was not possible to isolate a pellet after centrifugation.

At a D/P of 20% (Figure 7b), an aDS of 7.7 was induced. Both, the sampled drug concentration after cooling down to RT and after immediate centrifugation were similar, with concentrations slightly above the thermodynamic solubility of the drug in the same medium. Concentrations between 150–170 μg mL^–1^ were obtained. Notably, the samples that were left to rest for 30 min did not change their appearance upon cooling down. After centrifugation, a pellet was isolated even if a delay of 25–30 min was employed. The isolated pellet was XRPD amorphous, as depicted in Figure 8. To investigate whether the same change in solution state that was observed for the 10% D/P samples would occur at a later time point, the remaining solvent shift media was kept for 12 h at RT. It was observed that within this time period, the solution turned from a turbid solution to a clear, opalescent state, characteristic of Soluplus at RT. Sampled concentrations after centrifugation were substantially higher, approaching the nominal concentration of spiked RO6897779.

At a D/P of 30 and 40%, an aDS of 10.4 and 14.5 was induced, respectively (Figure 7c,d). Kinetic solubility profiles were comparable to the ones obtained for the 20% D/P samples. Redissolution of the amorphous phase was neither observed within the 30 min nor within the 12 h time period.

A comparison with the reference pattern of RO6897779 Form I demonstrated that each obtained pellet, independent of the D/P ratio, was amorphous after the 4 h solvent shift experiment. Visual characteristics of the pellet obtained for the 30% D/P sample are shown in Figure S5.

#### Qualitative Analysis of Colloidal Species via Multi-Angle Dynamic Light Scattering

**Figure 9:**
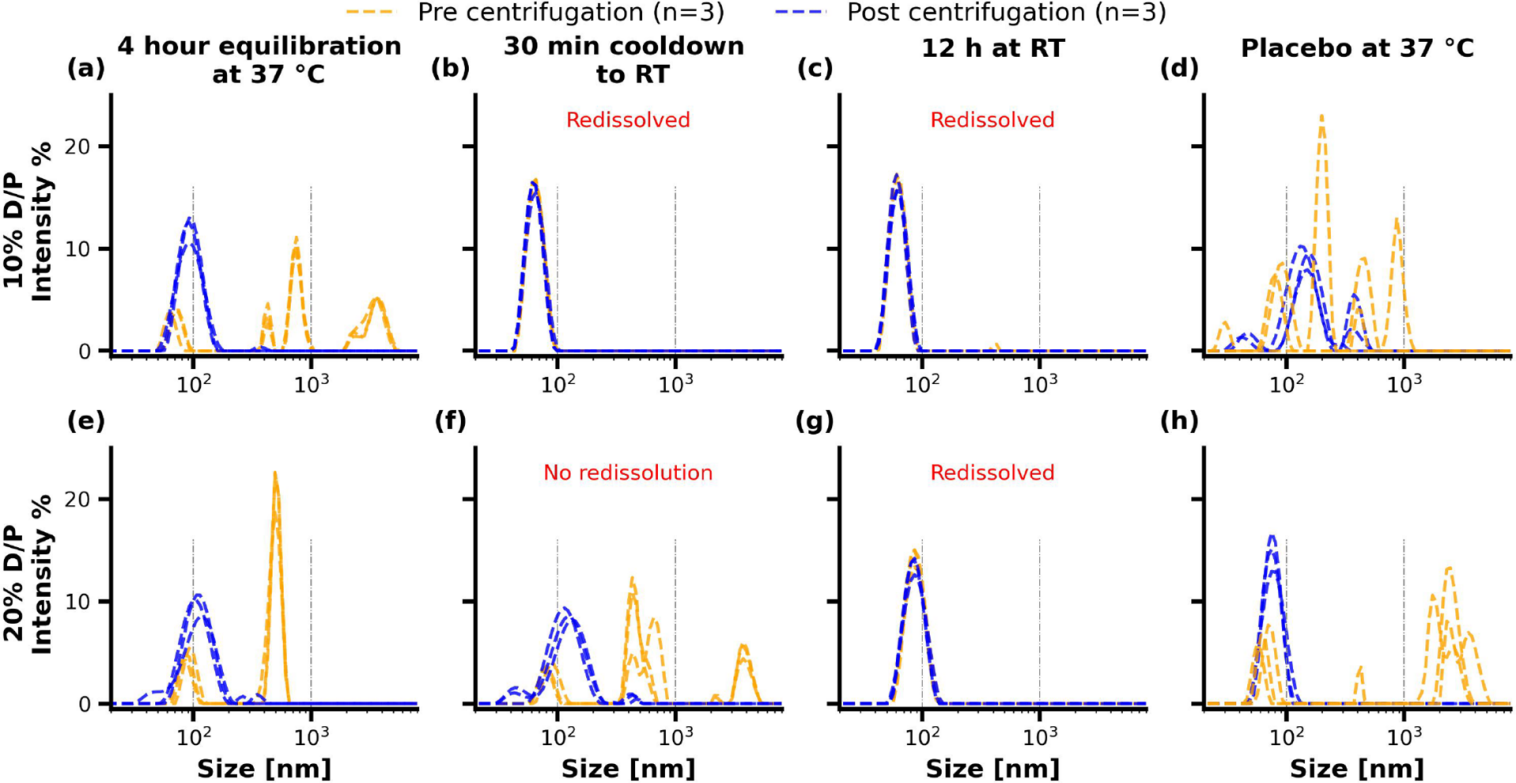
Qualitative comparison of size distributions of solvent shift media. (a–d) Size distributions for a 10% drug-to-polymer ratio and (e–h) size distributions for the 20% drug-to-polymer ratio. The samples were analyzed by multiangle dynamic light scattering (MADLS) pre- and post-centrifugation at either RT or 37 °C at different time points.

MADLS was conducted to further characterize the phase behavior of Soluplus in the absence and presence of the drug. Additionally, it was tested whether centrifugation of the media would isolate phase-separated Soluplus and it was examined whether such phase redissolved over time after cooling the samples down to RT. The samples were analyzed after the 4 h solvent shift experiment and at 30 min and 12 h after cooling the media down to RT. The results of the MADLS measurements pre- and post-centrifugation at D/P ratios of 10 and 20% are depicted in Figure 9.

At a D/P ratio of 10% (Figure 9a), the samples demonstrated highly multimodal size distributions prior to centrifugation. Analyzing the media after centrifugation revealed a monomodal size distribution at approximately 90 nm. At the same time point, the placebo vehicle demonstrated comparable behavior with significant multimodality prior to centrifugation, in contrast to post-centrifugation (Figure 9d). After the spiked solvent shift media was cooled down to RT over 30 min, the media exhibited one monomodal size pre- and post-centrifugation. It should be noted that this sample corresponds to the aliquots for which high concentrations were sampled during the solvent shift experiment by HPLC, as depicted in Figure 7a. The size distribution was analyzed again after 12 h, which did not result in a further size change. Although no solid particles were observed during the applied intermissions to cool the samples down to RT, potential sedimentation of particles was accounted for by agitating the solution prior to MADLS analysis of the noncentrifuged media.

Figure 9e–h presents size distributions by intensity for the 20% D/P ratio samples. The samples exhibited multimodality before centrifugation in the drug-loaded and placebo samples at 37 °C, which is less pronounced post-centrifugation. Contrary to the 10% drug load, the samples did not exhibit a monomodal size distribution after cooling the samples down to RT for 30 min pre-centrifugation. Post-centrifugation, the samples approximated a monomodal size distribution. Letting the samples rest for 12 h resulted in a monomodal size distribution pre- and post-centrifugation, analogously to the 10% D/P ratio after 30 min at RT. A pilot study was conducted, in which the drug content of these samples was reanalyzed by HPLC. The concentration of RO6897779 was considerably higher than the drug concentration of the samples during the 4 h solvent shift experiment (Data not shown).

#### Preparation and Characterization of Binary Amorphous Solid Dispersion

The spray drying of RO6897779 and Soluplus with a 10% DL was successful and yielded a fine, white, fluffy, and slightly static powder after the completion of a second drying step. The spray drying procedure gave an acceptable yield of 80%, which may be improved upon further process optimization. Based on SEM inspection, the powder was shown to be made up of deflated particles with a dimpled spherical shape, as seen in Figure S8. The average particle size distribution was found to be 6.96 ± 3.41 μm, determined from the measurement of 42 observed particles. Using headspace gas chromatography, it was confirmed that a solvent content lower than 1000 ppm remained in the sample, which complies with the specifications of ICH guideline Q3C (R5) for methanol. The LE of the spray-dried material was calculated to be 101.8 ± 0.3%. The material was analyzed before use via XRPD and DSC analysis, which confirmed the amorphous nature of the material by the absence of any Bragg peaks in the XRPD diffractogram and lack of a melting endotherm during heating in DSC (Figure S7). Upon reheating the material using DSC, a *T*_g_ of 78.59 ± 1.14 °C was measured.

#### Powder Dissolution of Binary Amorphous Solid Dispersion and Physical Mixture

**Figure 10:**
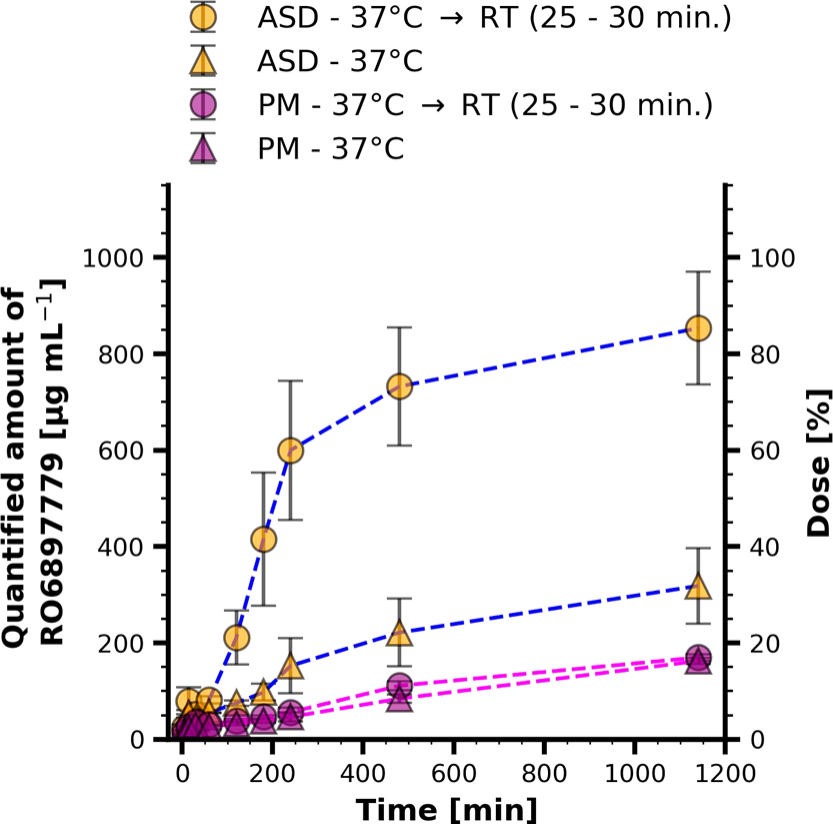
Powder dissolution results for a 10% [w/w] drug load binary amorphous solid dispersion (ASD) and a physical mixture (PM) at the same drug loading. Samples were filtered through centrifugal filter units at 11,500 *g* for 5 min through a filter membrane of 0.45 μm porosity. One aliquot was sampled immediately after centrifugation and another one after cooling it down to RT.

To demonstrate the practical implications of the phase separation behavior of Soluplus in FaSSIF-V1 at 37 °C, a non-sink dissolution test of a binary ASD at a drug load of 10% [w/w] was conducted. Complementary, a physical mixture of the same drug load was studied to assess the dissolution rate and solubility gain facilitated by the ASD. Dissolution of the ASD material was examined over an extended time period of up to 20 h to ensure complete dissolution. A depiction of the obtained dissolution profiles is provided in Figure 10.

Dissolution of the amorphous solid dispersion was very slow, and the data obtained by immediate centrifugation suggest that incomplete dissolution was obtained with around 30% of the drug being apparently dissolved after 20 h. This corresponds only to a marginal increase over the analyzed physical mixture. Crystalline precipitation as a cause for this behavior was ruled out due to the absence of crystallinity observed during the dissolution experiment via polarized light microscopy.

Analogously to the solvent shift experiments, a 30 min intermission was applied after centrifuging the sample through a filter membrane. The quantified amount of RO6897779 differed substantially depending on the sampling protocol during dissolution testing of the binary ASD. It is not expected that the difference in sampled drug concentration originates from undissolved ASD material, as this fraction would have been retained by the 0.45 μm PVDF filter membrane. Instead, phase-separated Soluplus containing RO6897779 was spun through the membrane due to liquid-like characteristics, as observed for placebo samples during NMR experiments. This phase redissolved upon temperature decrease within the 30 min intermission.

The concentrations sampled for the physical mixture are comparable and independent of the sampling protocol.

## Discussion

The successful development of ASDs is highly dependent on the selection of an appropriate polymer to ensure physical stability, as well as luminal supersaturation and the maintenance thereof during intestinal transit.^6,11,42^ Soluplus has gained considerable attention, due to its relatively unique attributes such as its amphiphilic character, facilitating drug solubilization through micelle formation, therefore acting as a kinetic and thermodynamic precipitation inhibitor.^15,17,43^ Another characteristic of Soluplus is its reported low LCST of 40 °C in water, which is currently less explored.^20^ At this temperature, a polymer solution begins to coacervate into a two-phase system, consisting of a polymer-rich and a polymer-lean phase.^22^ It has been shown that the presence of additives can modify polymer hydration and, as a result, change the LCST.^30,32,33,35,44^ However, these trends have primarily been related to ASD hydration and consequently dissolution rather than to the colloidal species present following the release of Soluplus under biorelevant conditions. However, a mechanistic understanding of the colloidal species present under these conditions is particularly relevant for comprehending in vitro*–*in vivo relationships of Soluplus-containing bioenabling formulations.

The data presented in this study report that, in addition to micelles, a polymer-rich colloidal phase existed in FaSSIF-V1, which was attributable to phase-separated Soluplus coexisting with polymeric micelles in solution at 37 °C. This finding challenges the common perception that Soluplus forms only micelles as colloidal species. The CP measurements confirmed this behavior, demonstrating that the LCST of Soluplus was considerably influenced by the media and polymer concentrations used (Figure 2). Comparisons of light transmission measurements in deionized water and FaSSIF-V1 demonstrated the influence of biorelevant media components on the CP, indicating that components in FaSSIF-V1 decreased polymer solvation. The influence of electrolytes on the CP of Soluplus has not been investigated in the context of biorelevant media components before. However, parallels can be drawn from previous works studying the dissolution kinetics of Soluplus-based ASDs in the presence of inorganic salts.^30,45^ It was shown that anions lower the CP along the Hofmeister series by acting as kosmotropic salts that may cause a “salting-out” of the polymer by affecting its hydration.^26^ It can be assumed that H_2_PO_4_^–^ contained in FaSSIF-V1 acted as a kosmotropic ionic species, causing a decrease in CP compared to deionized water. Shi et al.^35^ determined salting-out constants for Soluplus using a range of different salts. NaH_2_PO_4_ was among the salts resulting in the most pronounced decrease in CP. The influence of NaCl is assumed to be less pronounced, as Cl^–^ anions are considered to act as chaotropic salts, which would result in an increased CP value.^46^ Additionally, cations are expected to have a less significant influence on CP than anions.^30,47^ Salt effects on polymer hydration may either be attributed to direct interactions with the polymer or alterations of the hydration shell around the polymer.^32,48^ Moreover, the sodium taurocholate and lecithin present in FaSSIF-V1 also warrants consideration, as an interaction between lecithin and Soluplus has been reported before,^34^ which may serve as another explanation for the change in CP. It was noted for cellulose derivatives that surfactants and electrolytes may cause a synergistic reduction in CP, and thus the LCST.^23,49^ Overall, the cloud points determined in FaSSIF-V1 demonstrated a non-linear increase as a function of polymer concentration. This trend may be attributed to increased self-association in more concentrated solutions, potentially leading to an adverse impact on the solvation of the polymer, which was also observed for other polymer systems during CP measurements.^50^

Centrifugation of FaSSIF-V1 containing different Soluplus concentrations at 37 °C revealed that the polymer-rich phase is separable from the bulk media under mild centrifugal forces (3000 *g*, 5 min) and that the phase separation of Soluplus is fully temperature-reversible (Figures 3, and S2). Such behavior is of particular relevance for an in vitro assessment of Soluplus-based amorphous solid dispersions, as it would be commonly expected that only undissolved, or in the case of supersaturating formulations, potentially precipitated material is separated from the bulk media during dissolution testing. However, MADLS measurements pre- and post-centrifugation (11,500 *g*, 5 min) at 37 °C suggested that for all Soluplus concentrations, a separation of the polymer-rich phase from micellar species was attained by conventional centrifugation protocols applied during dissolution testing (Figure 4). The separated phase exhibited the characteristics of a high-density, viscous liquid. Quantitative ^1^H NMR spectroscopy revealed that in all cases, a substantial amount of polymer was separated. It was demonstrated that there is not a uniform concentration at which the polymer begins to phase separate across different polymer concentrations. This behavior was underscored by the CP measurements, where a dependency between polymer concentration and CP was noted, which may be indicative of increased self-association at elevated polymer concentration, leading to decreased solvation of the polymer.

**Figure 11:**
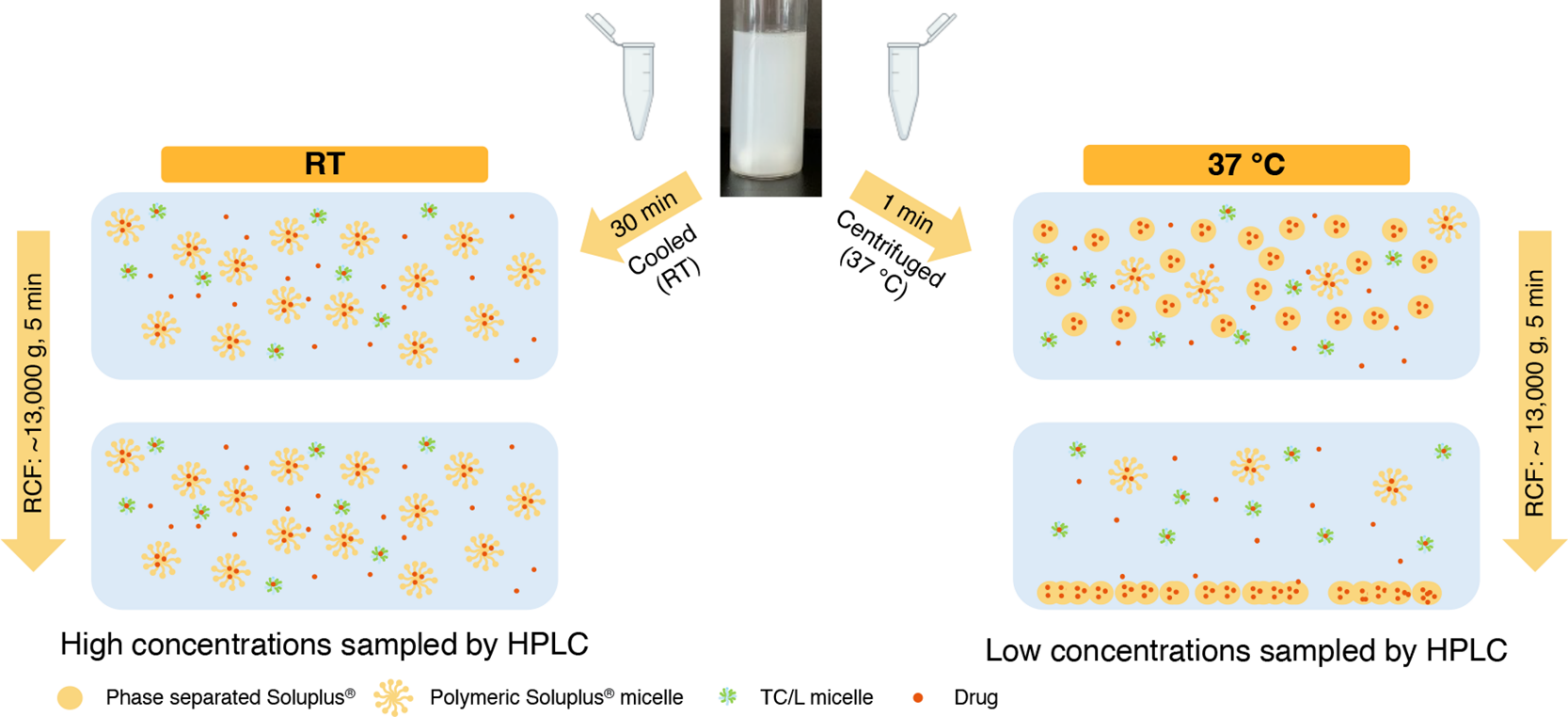
Schematic representation of the phase behavior of Soluplus and RO6897779 incorporation into formed colloidal species at a drug-to-polymer ratio of 10%. Drug containing polymer-rich phase was spun down by centrifugation if the sample was centrifuged immediately and not cooled to room temperature (RT). TC/L denotes sodium taurocholate and lecithin mixed micelles. Interactions between bile salts and phospholipids with Soluplus, as well as Soluplus monomers are not shown for clarity of presentation.

The identification of a separate colloidal species of Soluplus that is distinct from Soluplus micelles warranted further investigation into the interplay of drugs with such a phase. Solvent shift experiments and the isolation of an amorphous viscous phase after centrifugation demonstrated that RO6897779 was incorporated into the polymer-rich phase. The underlying behavior for the sample with a D/P of 10% is illustrated in Figure 11. Immediate centrifugation at all D/P ratios resulted in concentrations substantially below the nominal drug concentration during solvent shift experiments and rendered an XRPD amorphous pellet. On the other hand, centrifugation after 30 min, allowing the media to cool to RT, resulted in nominal drug concentrations at 10% D/P. Such behavior can be linked to the temperature-reversible phase separation of a polymer-rich Soluplus phase and by the characteristics of the isolated pellet. For a conventional amorphous precipitate, either unchanged apparent solubility or a decrease in apparent solubility would be expected instead of an increase, as the amorphous form constitutes the highest energy solid. It would rather be expected that a thermodynamically more stable form would precipitate according to Ostwald.^51^ However, upon decreasing the temperature, phase-separated Soluplus containing RO6897779 redissolved, which led to high sampled drug concentrations. This behavior was further confirmed by MADLS measurements conducted on the solvent shift media. For higher D/P ratios, cooling did not result in increased apparent drug concentrations, even though a polymer-rich phase of Soluplus containing RO6897779 was present. This may be attributed to the higher lipophilicity of such particles due to the higher fraction of the drug incorporated, which could impede redissolution during the intermission period. However, upon determining the concentration of RO6897779 after 12 h for the 20% D/P sample, substantially higher concentrations were obtained, suggesting redissolution of such colloidal structures over a longer time frame. For the 30 and 40% D/P ratio samples, the behavior was similar although at these ratios no redissolution even after 12 h was observed. It can be assumed that the miscibility of the drug with the wet polymer drives the drug incorporation into the phase-separated polymer. An assessment of drug–polymer interactions by the construction or calculation of ternary phase diagrams (water, drug, and polymer) may provide an explanation as to what physicochemical properties drive the incorporation of drugs into phase-separated Soluplus.^52^

More importantly, the investigated phase separation behavior demonstrated application to binary ASDs at a drug load of 10% [w/w]. Overall, the release of RO6897779 was very slow, and thus, a longer dissolution time was applied to study the phase behavior. A substantial difference between concentrations following the two sampling procedures for the ASDs was noted, which is not thought to be attributed to the undissolved ASD material. Since a centrifugal filter unit was employed, it is not expected that solid ASD particles escaped the filter membrane but that liquid, phase-separated Soluplus containing RO6897779 was spun through the filter membrane and redissolved upon cooling the samples down to RT. This behavior is the likely cause for the concentration discrepancies noted for both sampling protocols. It can be assumed that there is a complex mass balance of partitioning equilibria among phase-separated polymer domains, polymeric micelles, and molecularly dissolved drugs.

It is widely assumed that the molecularly dissolved drug fraction is the main driver for absorption and that apparently dissolved states may act as a reservoir, maintaining concentrations at the amorphous solubility of the drug intraluminally.^53^ In this regard, phase-separated drug-rich domains by liquid–liquid, and glass–liquid phase separation (LLPS/GLPS) have recently seen a surge of interest due to their potential to maintain a plateau in flux in vitro, and by demonstrating a positive influence on bioavailability in vivo.^54−56^ Drug-rich colloids are commonly considered as apparently dissolved. They are amorphous and exhibit, depending on their wet glass transition temperature, liquid characteristics, or glassy characteristics. Recent works by Ueda and Taylor^57^ demonstrated that surfactants and polymers may diffuse in and out of such drug-rich domains, which has been shown to reduce membrane transport of ketoprofen by a reduction in thermodynamic activity. The results of this study suggest that for Soluplus-based formulations phase separation may be excipient-driven, which is different from the common perception that phase separation from ASDs is triggered primarily by drug concentrations exceeding their amorphous solubility advantage. Similarly, the drug may diffuse into the reported Soluplus-rich phase.

Parallels can be drawn from hydroxypropyl methylcellulose acetate succinate (HPMC-AS), an ionizable polymer, for which a grade-dependent formation of nanoaggregates has been reported.^58−60^ The polymer is only sparingly soluble, even under intestinal conditions, which due to its charged nature leads to the formation of nanoaggregates in solution. Friesen et al.^58^ reported that such nanostructures may incorporate drugs, yielding a high-energy, amorphous state that can sufficiently stabilize drugs for several hours or even up to days in aqueous solutions. The frequently observed high apparent supersaturation and bioavailability of HPMC-AS-based ASDs was partly attributed to the redissolution of these structures, thus replenishing high molecularly dissolved drug concentrations. While the phase separation of Soluplus rendered particles of different sizes, the underlying concept demonstrates similarities, and further biopharmaceutical assessments are required to elucidate their influence on oral bioavailability.

Another aspect of the presented data is the development of suitable dissolution methods for Soluplus-based formulations. Thiry et al.^61^ reported a considerable influence of dissolution media and apparatus on obtained dissolution results. Based on the presented data, it could be hypothesized that a change in the LCST of Soluplus dependent on the media may provide an explanation for such behavior going forward. Previous research examined the dissolution kinetics of Soluplus-based amorphous solid dispersions containing tadalafil, revealing significant differences in dissolution profiles between fasted-state simulated gastric fluid and phosphate buffer (pH 7.2). These differences were attributed to the varying hydration of Soluplus, as tadalafil remains un-ionized within the investigated pH range.^45^ Additionally, an investigation of the more physiologically relevant bicarbonate buffer may provide merits for Soluplus-based ASDs, as salt-dependent hydration differences may have an especially relevant influence on attaining meaningful in vitro-in vivo relationships. It is thus recommended to study the phase separation behavior of Soluplus in commonly employed dissolution media, both biorelevant and non-biorelevant, to understand media-dependent changes in polymer hydration, which may significantly bias the obtained results.

## Conclusions

This study reports that aside from Soluplus micelles, a polymer-rich coacervate phase is present in FaSSIF-V1 at 37 °C. This colloidal species was characterized as a high-density liquid phase that can be spun down by centrifugation. The reason for this phase behavior was attributed to the LCST of Soluplus, which is lowered in the presence of biorelevant media components. This behavior was observed for a wide range of polymer concentrations, in both the absence and presence of the drug.

The results are of practical relevance, as drugs may interact and may be incorporated in such a phase. This polymer-rich, drug-containing phase is an important consideration in establishing meaningful in vitro*–*in vivo relationships. Further research needs to be conducted to understand how such a phase behavior influences oral bioavailability.

## Supporting Information Available

Numerical summary of the calculated cloud points; diagram of calculated cloud points for Soluplus dissolved in FaSSIF-V1 as a function of temperature, demonstrating an exponential decay; visual characteristics of centrifuged Soluplus after cooling the media down to RT, as well as sedimentation of unagitated Soluplus solutions/emulsions in FaSSIF-V1 at 37 °C; calibration curve established to quantify Soluplus using ^1^H NMR; and solid state characterization using XRPD on residual solids after equilibrium solubility experiments (PDF)
