# Supplementary material for: Mechanistic Investigation into the Phase Separation Behavior of Soluplus in the Presence of Biorelevant Media
Source: Mol Pharm. 2025 Mar 11;22(4):1958–72. doi: 10.1021/acs.molpharmaceut.4c01140 (PMC11979885; doi:10.1021/acs.molpharmaceut.4c01140)
Supplement: Supplementary file 1 — mp4c01140_si_001.pdf [file mp4c01140_si_001.pdf]

Mechanistic Investigation into the Phase Separation Behavior of Soluplus® in the Presence of Biorelevant Media

Justus Johann Lange,[†,#] Malte Bøgh Senniksen,[‡,¶,#] Nicole Wyttenbach,[§] Susanne Page,[‡] Lorraine Bateman,[†,∥] Patrick J. O'Dwyer,[†] Wiebke Saal,[§] Martin Kuentz,[⊥] and Brendan T. Griffin[*,†]

†*School of Pharmacy, University College Cork, College Road, Cork, T12 R229, Cork County, Ireland*

‡*Roche Pharma Technical Development, Roche Innovation Center Basel, F. Hoffmann-La Roche Ltd., Grenzacherstrasse 124, Basel, CH-4070, Switzerland*

¶*Fraunhofer Institute of Translational Medicine and Pharmacology, Theodor-Stern-Kai 7, Frankfurt, DE-60596, Germany*

§*Roche Pharma Research and Early Development, Therapeutic Modalities, Roche Innovation Center Basel, F. Hoffmann-La Roche Ltd., Grenzacherstrasse 124, 4070 Basel, Switzerland*

∥*University College Cork, Analytical & Biological Research Facility, College Road, Cork, T12 YN60, Ireland*

⊥*University of Applied Sciences and Arts Northwestern Switzerland, Institute of Pharma Technology, Hofackerstrasse 30, Muttenz, CH-4132, Switzerland*

#*These authors contributed equally to this work.*

E-mail: brendan.griffin@ucc.ie

Supporting Information Available

Table S1 lists the cloud points calculated based on the light transmission over temperature profiles for different Soluplus® concentrations dissolved in FaSSIF-V1 and deionized water, as illustrated in Figure 2.

Table S1: Calculated cloud points defined as 50% light transmission of Soluplus® dissolved in FaSSIF-V1 and deionized water for different concentrations.

Soluplus® concentration	FaSSIF-V1	Deionized water
$9.00\,\mathrm{mg\,mL^{-1}}$	34.21 °C	39.07 °C
$4.00\,\mathrm{mg\,mL^{-1}}$	35.07 °C	n.a.
$2.33\,\mathrm{mg\,mL^{-1}}$	36.44 °C	n.a.
$1.50\,\mathrm{mg\,mL^{-1}}$	39.98 °C	n.a.

Figure S1 illustrates the exponential decay in cloud point as a function of polymer concentration.

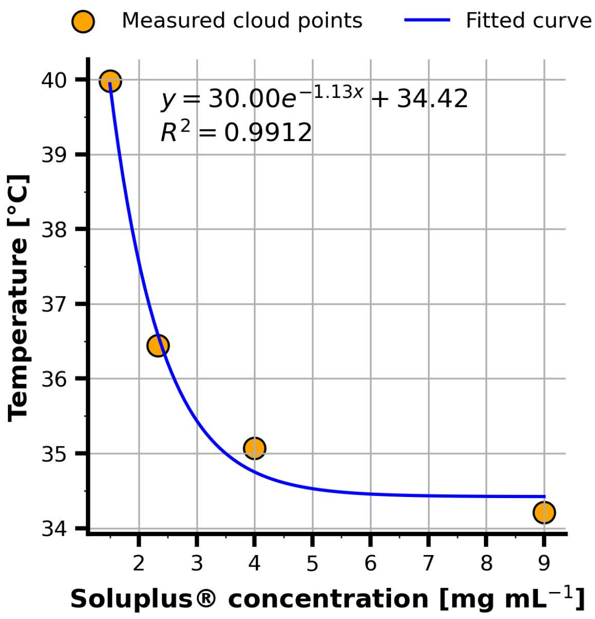

Figure S1: Exponential decrease in cloud point as a function of Soluplus® concentration in FaSSIF-V1.

Figure S2 depicts the centrifuged Soluplus® media dissolved in FaSSIF-V1 after cooling it down for 10 min to RT.

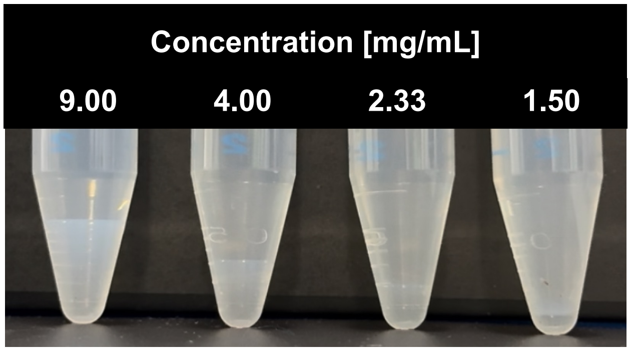

Figure S2: Centrifuged Soluplus® dissolved in FaSSIF-V1 after letting the sample cool down to room temperature (RT) within 10 min.

Figure S3 depicts the sedimentation of phase separated Soluplus® dissolved in FaSSIF-V1 after three hours of equilibration at 37 °C.

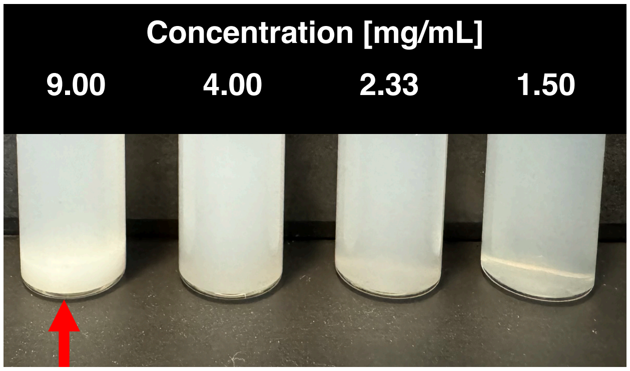

Figure S3: Unagitated Soluplus® dissolved in FaSSIF-V1 observed at 37 °C after three hours of equilibration. The nominal Soluplus® concentration was dissolved at RT before equlibration at 37 °C.

Figure S4 depicts the established calibration curve based on the ratio of the PEG6000 signal of Soluplus® over the internal reference standard TMSP-d_4.

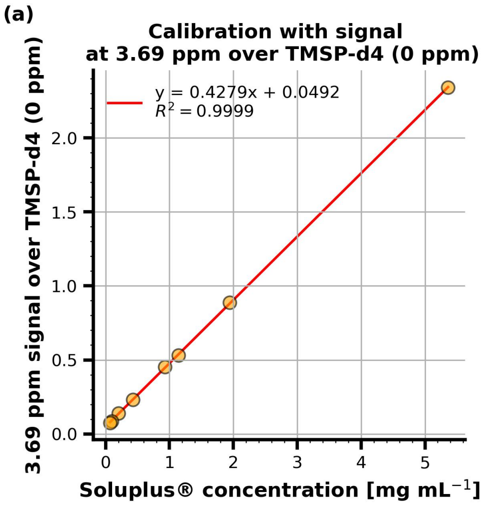

Figure S4: Calibration curve obtained by integrating the signal at 3.69 ppm corresponding to PEG6000 over TMSP-d_4.

Figure S5 shows the isolated pellets from the solvent shift experiment at 37 °C at a drug to polymer ratio of 30%.

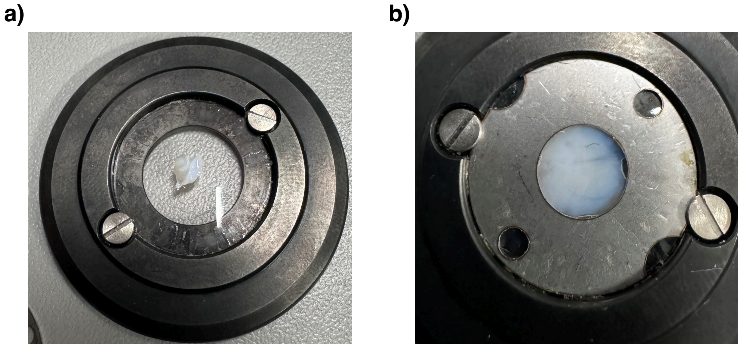

Figure S5: Pellet isolated from solvent shift media at a drug to polymer ratio of 30% by centrifugation. The pellet exhibits viscous characteristics and does not consist of separate particles.

Figure S6 demonstrates the absence of solid form changes after 48 hours of equilibrating by comparison to untreated RO6897779 Form I.

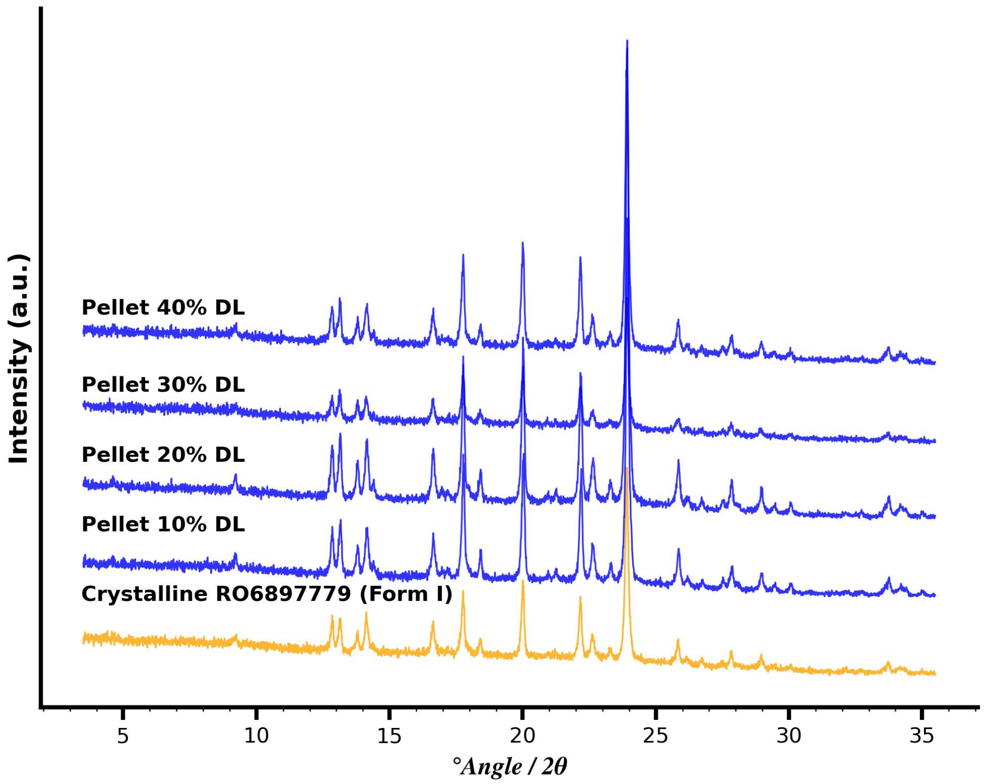

Figure S6: XRPD patterns of residual solid after the 48 hour solubility experiment.

Figure S7 illustrates the determined solid state characteristics of the spray dried material and the crystalline reference material of RO6897779.

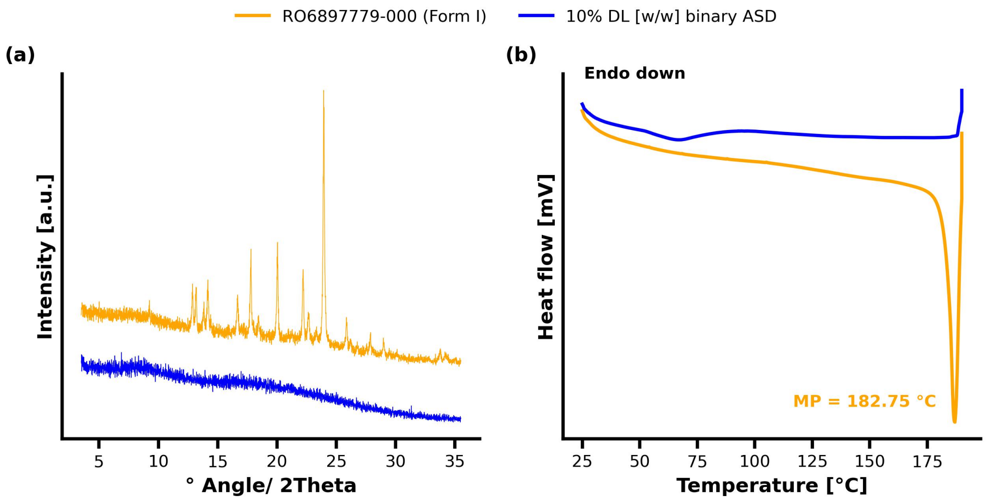

Figure S7: a) X-ray powder diffraction (XRPD) patterns, and b) Differential Scanning Calorimetry (DSC) enthalpograms for the manufactured spray dried dispersion, and crystalline RO6897779 Form I.

Figure S8 depicts the SEM images of the spray dried material.

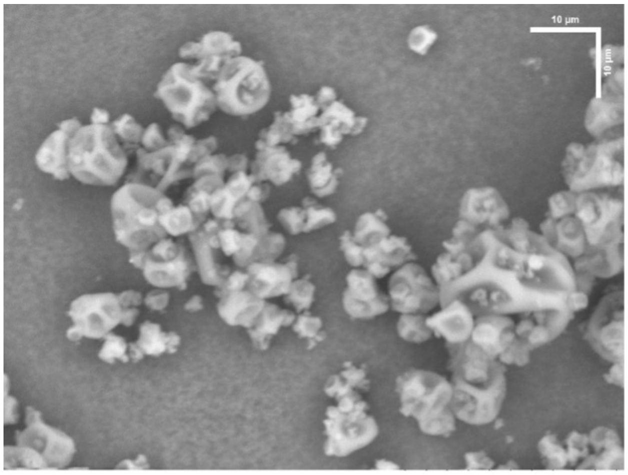

Figure S8: Scanning electron microscopy images of RO6897779-Soluplus® amorphous solid dispersion particles at a drug load of 10 % manufactured by spray drying. The scale bar represents 10 µm in vertical and horizontal orientation and the sample was magnified 2000-fold.
